# On-Demand Information Retrieval in Sensor Networks with Localised Query and Energy-Balanced Data Collection

**DOI:** 10.3390/s110100341

**Published:** 2010-12-30

**Authors:** Rui Teng, Bing Zhang

**Affiliations:** National Institute of Information and Communications Technology, Kyoto 619-0288, Japan; E-Mail: zhang@nict.go.jp

**Keywords:** on-demand information retrievals, localised data query, data collection, attribute-object name, sink proxy, balancing of energy consumption

## Abstract

On-demand information retrieval enables users to query and collect up-to-date sensing information from sensor nodes. Since high energy efficiency is required in a sensor network, it is desirable to disseminate query messages with small traffic overhead and to collect sensing data with low energy consumption. However, on-demand query messages are generally forwarded to sensor nodes in network-wide broadcasts, which create large traffic overhead. In addition, since on-demand information retrieval may introduce intermittent and spatial data collections, the construction and maintenance of conventional aggregation structures such as clusters and chains will be at high cost. In this paper, we propose an on-demand information retrieval approach that exploits the name resolution of data queries according to the attribute and location of each sensor node. The proposed approach localises each query dissemination and enable localised data collection with maximised aggregation. To illustrate the effectiveness of the proposed approach, an analytical model that describes the criteria of sink proxy selection is provided. The evaluation results reveal that the proposed scheme significantly reduces energy consumption and improves the balance of energy consumption among sensor nodes by alleviating heavy traffic near the sink.

## 1. Introduction

Recent progress in wireless sensor networks has revealed that multifarious sensors can be used to monitor various environment objects, such as plants, farm soil, factory instruments, and bird nests. To support ubiquitous computing, sensor networks should be integrated with the Internet, enabling people around the world to ubiquitously access information about the physical world [1]. In wireless sensor networks, sensor nodes can either periodically report sensing data to a server in a proactive manner, or deliver sensing data on-demand, namely when a user queries the sensor nodes only. On-demand information retrieval enables interaction between Internet users and sensor nodes, and lets a user retrieve up-to-date sensing information (such as the up-to-date landscape of a region) [1–4].

On-demand information retrieval consists of a data query phase and a data collection phase. A user initiates the process by sending a query message to a sensor network through the sink node, which broadcasts the query message to nodes in the network. The destination of a user’s query is not described by the sensors’ identifiers (IDs) but by the users’ interest, as represented by the name of data attributes [2]. For instance, a typical query would not be destined to a certain sensor node for its temperature data, but rather to the corresponding sensor nodes for ‘the temperature in the north-west quadrant’. Upon receiving the query message, the sensor nodes that match the query deliver sensing data to the sink node. The sink node then sends to the user the collected sensing data via the Internet.

Because of the power constraints of battery-powered sensor nodes, energy efficiency is a substantial requirement of information retrieval in sensor networks. For this reason, each query message should be sent to sensor nodes with small traffic overhead and sensing data should be collected with low and balanced energy consumption. However, on-demand query messages are generally forwarded to sensor nodes in network-wide broadcasts, which create a large traffic overhead. Although geocasting can be used to localise a query dissemination area, the success of conventional query geocasting relies on two assumptions: the user specifies a region as the query’s designated area and the specified query region includes all sensors that are corresponding to the query [5,6]. In fact, a user who issues a query to sensor networks often knows only the attributes of his/her interests, such as temperature information, and the name or location of relevant query objects such as a room. The location of a query object that the user knows may also be different from the location where the relevant sensors are deployed.

Sensing data collection has meanwhile been the subject of extensive research. Data aggregation is a key solution to achieve energy efficiency. To aggregate sensing data in the sensor network, various network structures such as clusters and chains are utilised. Most of these data collection structures are applied to the proactive data collection, in which sensors periodically report data to a data server [7–11]. Note that on-demand information retrieval initiates query and data collection at arbitrary times from users with diverse interests. This makes it difficult to utilise data aggregation structures of clusters or chains, because of the complexity of setup and maintenance of the network structures of data aggregation.

Many conventional on-demand data collection approaches adopt reverse trees, which are built based on the query dissemination. Sensing data are delivered in the tree towards the sink node, which is the root of the tree. However, this on-demand data collection is inefficient because of the large and unbalanced energy consumption in the network. Because most data are accumulated at a sink node with multihop relays, nodes near the sink node are likely to consume more energy than nodes remote to the sink node.

This paper addresses the problem of how to localise query dissemination and maximise data aggregation in an on-demand manner. The goal of the proposed approach is not only to reduce total energy consumption of information retrieval in sensor networks, but also to balance energy consumption among individual sensor nodes. We propose an approach of localised on-demand information retrieval that explores the name resolution for each query before the query message is forwarded to sensor nodes. The name resolution resolves a query name to the addresses of sensors that are corresponding to the query, leading to node-wise information retrieval in sensor networks. According to the location of each sensor that corresponds to the query, the area where the resolved sensors reside can be specified. To localise query dissemination and maximise in-network data aggregation, we propose the use of sink proxies as local query broadcasters and local data collectors. Each sink proxy is selected among sensor nodes according to the name resolution result.

The specific features of this paper are as follows: first, an attribute-object naming system and query resolution operation are adopted in the on-demand information retrieval. The query resolution, which could be considered analogous to the function of the Domain Name System (DNS) in the Internet, resolves a query’s name to local node IDs, the locations of a collective of corresponding sensors, and sink proxies, before the query message is disseminated to the sensor network. Second, we adopt a localised on-demand information routing scheme that consists of two parts: Localised Data Query Distribution (LDQD) and Localised Sensing Data Collection (LSDC). LDQD and LSDC significantly reduce the energy consumption of information retrieval. Given consideration of the temporal-spatial nature of user queries, LDQD and LSDC adopt dynamic sink proxies based on query content to improve the balance of energy consumption among sensor nodes. Third, to effectively select a sink proxy for each query, an analytical model is provided to describe the criteria of sink proxy selection. Both energy efficiency and energy balance are considered to effectively choose and utilise sink proxies. Fourth, this paper extensively studies the performance of the proposed approach for localised data query and collection through simulation evaluations of the NS-2 simulator integrated with the IEEE 802.15.4 module [12,13]. In the simulation, we investigate protocol performance in terms of both total energy consumption and the distribution of energy consumption among individual sensor nodes.

The remainder of this paper is organised as follows. Section 2 introduces the related work. Section 3 introduces the procedures for a proposed approach to localised information retrieval. Section 4 describes the system analysis of the proposed approach. Section 5 describes the evaluation and numerical results of the proposed approach and Section 6 concludes.

## 2. Related Works

Information retrieval in sensor networks can be broadly categorised into two types: proactive and on-demand. In proactive information retrieval, sensor nodes periodically report data with a predefined data rate and data delivery infrastructures. Meanwhile, in on-demand information retrieval, a query is delivered to the sensor network and data are collected according to the query’s requirements.

Existing approaches for proactive information retrieval mainly focus on the energy-efficient collection of sensor data [7–11,14,15]. To aggregate sensing data, sensor nodes are organised into infrastructures, such as clusters, chains or optimised trees. For example, LEACH (Low-Energy Adaptive Clustering Hierarchy) is a prevailing scheme of proactive data collection based on a dynamic clustering approach [7]. In LEACH, data can be efficiently collected and aggregated in a hierarchical way. EEDC (Energy Efficient Data Collection) is another proactive data collection approach based on a cluster infrastructure [9]. It introduces an energy-efficient data collection approach by exploiting spatiotemporal correlation of sensing data. Energy efficiency is achieved by reducing the spatial sampling rate of sensor nodes. PEGASIS (Power-Efficient Gathering in Sensor Information Systems) organises sensor nodes into a chain structure rather than clusters [15]. The network structures of data aggregation, such as clusters and chains, significantly improve the energy efficiency of data collection. However, generating and maintaining a network structure entail much additional cost. In addition, a fixed network structure for data collection creates an energy imbalance. That is, the nodes near the sink node or cluster head generally consume much more energy than other nodes do.

Unlike proactive data collection, query-based on-demand information retrieval generally has a temporary organization of sensor nodes to collect data. A straightforward approach for on-demand information retrieval generally utilises a broadcast-reverse tree based model. A query is broadcast from the sink to the network and then a tree structure is constructed together with the task allocation of query. Sensors report their data back to the sink using the tree structure. Collected data can be aggregated on the tree. Most on-demand information retrieval approaches follow this model, but they differ in the mechanisms they use for query propagation and route selection for data collection [16–19].

Efficient query propagation is considered in [16]. The target is to propagate query message to a limited number of nodes so as to save energy. The ideal case is the minimized tree that includes no redundant broadcast of query message. However, this tree-based query requires repetition of sink queries and is not suitable to the arbitrary queries from users.

If a query specifies a query area, a spatial query broadcast can be archived based on geocasting or multicasting, which reduce redundant traffic overhead and achieve energy efficiency [5,20]. A typical approach Location based multicast (LBM) can be used to efficiently broadcast query messages in on-demand information retrieval in the case that there is a predefined region as the query destination [20]. LBM uses a multicast zone that is rectangular in shape and contains both the sender and all destination nodes. LBM assumes a roughly predefined region in which destination nodes reside. But the predefined region of destined sensor nodes might be either large or not available to a user who initiates on-demand information retrieval.

Although geocasting can be used to localise a query dissemination area, these operations rely on the assumptions that the user specifies a region as a query designated area. In fact, a user-defined region of destined sensor nodes may not always be available to a user who initiates on-demand information retrieval, or it may differ from the area that contains the sensor nodes corresponding to the query. There are other location based approaches that attempt to achieve localised routing operations in ad-hoc networks, however, most of these approaches focus either on end-to-end routing issues or only on the dissemination of a message to a predetermined area [5].

The route(s) for a sensor node to reply with data can be selected based on various criteria, for example, using a small hopcount in [21–23], minimum energy cost of data delivery in [17], adopting reliable routes from multiple candidates to avoid route failure in [3], or using an alternative path obtained from neighbouring nodes to improve security [24]. The reverse-tree-based data collection is simple to construct and imposes little additional overhead, as it is built on the basis of query dissemination. In contrast, a data collection tree with a root at the sink causes most of the collected data to be aggregated at the sink, and nodes near the sink experience heavier traffic than nodes remote from the sink, draining their energy faster and leading to network faults even though there are many nodes with substantial residual energy.

A tour based data collection approaches is proposed in [25]. The aim of this approach is to save energy consumption in data collection and query propagation. The sink builds a source route as a tour for data collection, and the source route tour guides the delivery of query together with data collection from sensors. In the data collection from small sensors, this approach is good to reduce energy. However, in case of data collection from a large number of nodes, the source route is a high cost of overhead for each sensor to handle. Further, the efficiency of source route is low and complex [26].

Mobile sink based data collection has been studied in recent years. The support of mobile sinks in sensor networks increases the flexibility of user interaction with sensor networks. The mobile sink can be a user’s mobile phone. It can be applied in case a user is in the sensor networks. The mobile sink alleviates the energy overuse at a certain place as that in the sensor networks with static sink [27,28]. On the other hand, the mobility of mobile sink introduces the routing complexity. Using mobile sink to fetch sensing data was considered in [29]. When sensing data is required, the mobile sink will go to the place near the sensor node, and direct collect sensing data by one-hop communication. This requires the high intelligent sink which can move automatically. This approach might be applied to the intelligent robot network.

Recent researches have tried to analyze the phenomenon of uneven energy consumption in sensor network [30–32]. One attempt to alleviate the problem of energy uneven consumption by using specialized node deployment is proposed in [30]. More nodes are deployed near the sink node to share the energy consumption. The demerit of this approach is that the specialized deployment also causes deployment redundancy.

This paper discusses the problems in on-demand information retrieval. The disadvantages of conventional on-demand information retrieval are summarized as follows: first, query messages are often broadcast throughout the network in a data-centric manner, without distinguishing the corresponding individual sensor node ID. These on-demand data queries generate an excessive amount of redundant network traffic and energy consumption in large sensor networks. Second, sensor nodes reply to queries by sending data back towards the sink node via various paths (such as the reversed paths obtained from query broadcasting), and most of the data are accumulated at a sink node. This causes high and unbalanced energy consumption, since nodes near the sink node are likely to consume more energy than nodes remote from the sink node.

## 3. Localised Information Retrieval with Query Resolution

### 3.1. Query Resolution Mechanism

A query resolution resolves the name of a query to the corresponding sensor nodes before the query message is forwarded to sensor nodes. A query resolution is somewhat analogous to the name resolution at the Domain Name System (DNS) widely used on the Internet. Domain name resolution is a process of resolving a host name to an IP address before a user sends the initial IP packet. A main function of domain name resolution is to facilitate the IP routing from the source node to the destination. Similarly, the query resolution mechanism in our scheme attempts to resolve each query’s name to facilitate efficient query dissemination and data aggregation in sensor networks.

**Figure 1.**
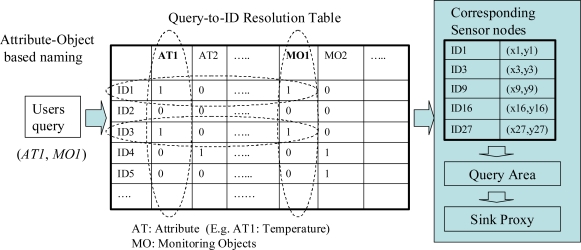
Query to IDs resolution.

Figure 1 illustrates an example of a query resolution process. We use an Attribute-Objects name (AO name) based on low-level naming, that describes the query’s name as well as the properties of sensor nodes. A user’s query can be specified by the interested objects and attributes of sensor data. For instance, in a building automation application, a query might be what temperature (attribute) status is in the storage room (object). In a factory application, a query could concern the vibration (attribute) status around the robot in a manufacturing line (object). In the same way, a sensor node can be described by the object that it is monitoring, and the physical attribute such as temperature or humidity that describes a sensor’s type.

A resolution table is adopted in the query resolution mechanism to discover the locality of sensor nodes corresponding to a query. The resolution table maps the attribute name of each query and sensing IDs. The query resolution table is implemented at the sink node of a sensor network. This is because the sink node generally has greater power and memory capacity than sensor nodes have. The query resolution table is initially achieved by the following two operations:

- Each sensor node registers its ID, location, sensing attributes and monitoring object (if the sensor knows it) with the sink node.
- A sink node constructs a table that maps sensing attributes and monitoring objects to sensor IDs.

The registration can operate in conjunction with the network configuration in the phase of network formation, minimising additional overhead. Because the sink node is generally a powerful node, it can maintain a large resolution table. Although the use of location information may increase the complexity of the system, the development of localization sin sensor networks in the past decades has provided more and more low cost, easily-maintained locating system [19,33,34]. Many applications of sensor networks use location of sensors to know the network deployment, obtain sensing context, analyze sensing data, and to perform network maintenance, recovery, and task management, *etc*. Because a general static sensor node is used in a sensor network, the requirements for updating the location of sensor node are minimal. The object that a sensor is monitoring refers to static objects that the sensors are monitoring over the long term, instead of the dynamic results that a sensor detected.

The sink node processes each query before it is disseminated to sensor nodes. As shown in Figure 1, when the sink node receives a query message, it resolves the name of the query (*i.e.*, AT1, MO1 in the example of Figure 1) to the corresponding sensor IDs (*i.e.*, node 1, 3, 9, 16, 27), according to the resolution table. After a query’s name is translated into an ID group corresponding to sensor nodes, the sink node calculates the query area by deriving a rectangle in which all corresponding sensor nodes reside. A rectangle area is calculated as follows, based on the locations (xi,yi) of each sensor node i that is in the ID list obtained from the query resolution:

(1) Rect=[min(x1,x2,…xn),min (y1,y2,…yn),max (x1,x2,…xn),max (y1,y2,…yn)]

where n is the number of corresponding sensor nodes.

**Figure 2.**
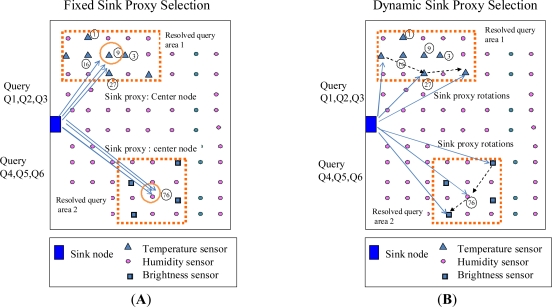
Two Types of Sink Proxy Selections.

A sink proxy that will be used for localised query broadcasts of queries and local data collections is then selected according to the query resolution result. As show in Figure 2, there are two types of sink proxy selection schemes. One is fixed selection scheme, in which an identical sink proxy (such as node 9 in the Figure 2 **A**) will be selected at different times for the queries (such as Q1, Q2, Q3 in Figure 2 **A**) that have the same query resolution result; the other is dynamic selection scheme, in which various sink proxies will be selected at different times for the queries that have the same query resolution result, as shown in Figure 2 (B).

As for the fixed selection scheme, a simple way adopted in this paper is to select a sink proxy that is the “centre node” of the query area. The centre node is defined as the node that is nearest the centre location of the query area. If the closest nodes are equidistant from the centre of the query area, the node with the smallest ID is chosen as the centre node. In Section 3.4 we discuss the dynamic sink proxy selection process in detail.

Consequently, the sink proxy provides the node information at which the query message starts to be broadcasted and the query area gives a region in which a query message can be disseminated. According to the calculated query area and sink proxy, the distribution of a query message is performed by two steps: unicast distribution and geocast distribution. These are introduced in the following subsection.

### 3.2. Localised Data Query Distribution with the Sink Proxy

Localised Data Query Distribution (LDQD) consists of two steps: Query unicast, and Query geocast. The unicast distribution is used to deliver query messages from a sink node to the sink proxy. The sink node calculates a source route to a sink proxy based on the nodes’ location. There are a number of potential approaches to building a source route. For example, the sink node first selects the node that is nearest the sink proxy among one-hop neighbours of the sink node. The selected node is included in the source route. In the same way, the sink node continues to calculate the next node in the source route, by selecting the node nearest to the sink proxy among the neighbours of the previous selected node in the source route. This process continues until a source route is found to the sink proxy. Similar approaches were elaborated in [35].

After the query message is delivered to the sink proxy in the query area, it is geographically broadcast to all sensor nodes inside the query area. The sink proxy forwards the query message to its one-hop neighbours. As illustrated in algorithm1, upon receiving the query message, each sensor node checks whether it should relay the packet to its neighbours by the following rules: a) Has not received this packet before; b) In the query area. If both a) and b) are satisfied, a sensor node will relay the packet to its neighbouring nodes. Otherwise, the sensor node just discards the packet.

### 3.3. Localised Sensing Data Collection with the Sink Proxy

**Figure 3.**
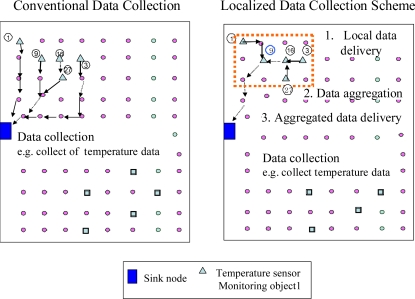
LSDC data collection.

Localised Sensing Data Collection (LSDC) consists of three steps: local data delivery, data aggregation, and aggregated data delivery to the sink node. An example of the LSDC scheme is illustrated in Figure 3. On receiving a query message, the corresponding sensor nodes send the sensing data back to the sink proxy in a local region. This is achieved using the reverse path obtained from the query geocast initiated by the sink proxy. The sink proxy, such as node 9 shown in Figure 3, collects the sensing data locally before forwarding it to the sink node. The sink proxy aggregates the sensing data by placing multiple sensing data into one packet. To efficiently aggregate sensing data and deliver the aggregated data to the sink node, the sink proxy sends the aggregated data according to the following two conditions: (a) The number of aggregated sensing data equals the largest number that the packet can contain; (b) The number of aggregated sensing data equals the number of the total corresponding nodes, which is calculated from the sink node. If either of these two conditions is satisfied, the sink proxy delivers the aggregated packet to the sink node. The sink proxy sends aggregated data to the sink node by unicasting, which can be obtained from the query dissemination. Finally, the sink node collects all data that correspond to the query. The collected data are then delivered to the user who initiated the query.

### 3.4. Balancing of Energy Consumption and Rotations of a Sink Proxy

Query content varies according to users’ interests, and the group of sensor nodes responding to a query varies with the query contents. This query diversity generally enables localised data collection to not only alleviate the overuse of energy at nodes near the sink node, but also to avoid overuse of energy at particular sensor nodes, such as sink proxies, achieving distributed energy consumption among sensor nodes.

In the case many users have same query interest at different times, a fixed sink proxy will result in an energy overuse at the sink proxy corresponding to these queries. This is because that the multihop relay features of wireless sensor networks require nodes near (in hop-distance) the sink proxy relay packets from nodes further away from the sink proxy [30,31]. To avoid such overuse of energy at a sink proxy, a rotation operation on the sink proxy is utilised. When a sink node receives a query that asks for the same content as that of the previous query, it will assign a new sink proxy randomly in the query area, in order to distribute the energy among the local sensor nodes corresponding to the query. We use LDQD-random and LSDC-random to generally represent the approaches that use randomly selected nodes as sink proxies for LDQD and LSDC.

## 4. System Analysis

In this section, we describe an analytical model of sink proxy selection. Furthermore, we analyze the impact of the proposed approach on the energy bottleneck in data collection. Here, we call the information retrieval model in our proposed approach the S3 (Sink-Sink_Proxy_Sensors) scheme, and call the conventional on-demand information retrieval model as S2 (Sink-Sensors).

### 4.1. Selection of Effective Sink Proxies

The effective selection of sink proxies is expected to result in both balanced and decreased energy consumption of compared with the conventional S2 retrieval model. Because the use of sink proxies enables maximum data aggregation among various sensor nodes, it alleviates the heavy energy consumption near the sink. Hence, if the use of each selected sink proxy also causes a lower energy cost compared with the conventional S2 information retrieval approach, the proposed approach then achieves both balanced and decreased energy consumption. Therefore, the basic criterion for the selection of a successful sink proxy is that both the following two conditions are satisfied: (a) The energy cost of the S3 query is no larger than that of conventional S2 query; (b) The energy cost of the S3 data collection is not larger than that of the conventional S2 data collection.

#### A. Data query cost and effective sink-proxies for the data query

For analytic tractability, we analyze S2 and S3 schemes in a simple setting. We assume the query area is a square consisting of N nodes. The average energy consumption of a one-hop transmission of a packet is assumed to be *E*_0_.

In the one-to-many model of an S2 query, flooding is typically adopted for disseminating a query to nodes in the network. The energy cost EQ2 of a data query is given by:

(2) $$E_{Q2}=N*E_{0}$$

In S3 information retrieval, in contrast, the operation consists of unicasting from sink to sink_proxy, and geocasting from sink_proxy to sensors in the geocast area, which is a combined rectangle area that contains both sink proxy and the query area. Given that b is the ratio of the node number in the geocast to the total node number N, and 0 < *b* ≤ 1. Therefore, *b* * *N* is the node number in the query geocast area of S3. Let *R_u_* be the hops of transmission in the unicast from the sink to the sink proxy. Then, the energy cost of S3 query can be computed as:

(3) $$E_{Q3}=b*N*E_{0}+R_{u}*E_{0}$$

As a result, the ratio *R_Q_* of query cost of S3 to S2 is given as follows:

(4) $$R_{Q}=\frac{E_{Q3}}{E_{Q2}}=\frac{b*N*E_{0}+R_{u}*E_{0}}{N*E_{0}}=\frac{b*N+R_{u}}{N}$$

The smaller *b* and *R_u_* are, the smaller *R_Q_* is. This means greater energy savings.

When a S3 query saves energy compared with the conventional S2 approach, the ratio of the S3 query cost to the S2 query cost is no larger than 1. That is:

(5) $$R_{Q}\leq 1$$

**Figure 4.**
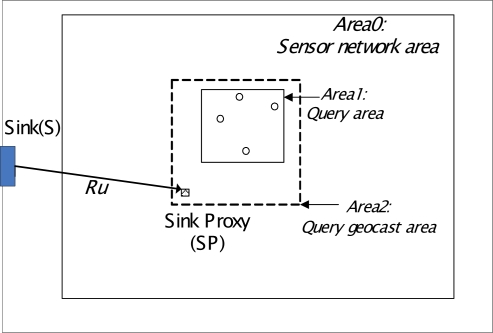
Selection of effective sink proxy for data query.

When *R_u_* ≤ *N*(1−*b*), we have *R_Q_* ≤ 1. Therefore, an effective sink proxy should enable *R_u_* ≤ *N*(1−*b*). If a query interest area is defined, the value of b and Ru can be determined by the position of a sink proxy. Given a sink proxy candidate with location coordinates of (x,y), and an resolved query area (Xmin, Xmax, Ymin, Ymax), as shown in Figure 4, we can determine whether the sink proxy candidate is a suitable sink proxy for S3 query geocasting by calculating b and Ru if the sink proxy candidate is used. The query geocast area in S3 model can be calculated as (X’min, X’max, Y’min, Y’max), where X’min = min(x, Xmin), X’max = max(x, Xmax), Y’min = min(y, Ymin), Y’max = max(y, Ymax). We can then determine the ratio R of query geocast area to the network area. R equals b, assuming nodes are uniformly deployed. Further, since Ru is the shortest hopcount from the Sink to the sink proxy candidate, it can be calculated according to the Greedy Perimeter Stateless Routing (GPSR) hop-by-hop routing process [26]. Consequently, *RQ* can be obtained.

If:

$$R_{Q}=\frac{E_{Q3}}{E_{Q2}}=\frac{b*N+R_{u}}{N}\leq 1,$$

then the sink proxy candidate can be selected as a sink proxy for S3 query geocasting.

#### B. Data collection cost and effective sink-proxies for the data query

In the many-to-one model of S2 data collection, the energy cost can be approximately given by the sum cost of the replied data unicast from each sensor node to the sink. Note *α* is the ratio of sensor nodes that will reply a query message, and *α* * *N* is the node number of sensor nodes corresponding to the query of S3. Let *R̅*_0_ be the average route length from each corresponding sensor node to the sink. Thus, the energy cost of S2 data collection, denoted by *E*_*C*2_, can be written as:

(6) $$E_{C2}=\alpha *N*E_{0}*{\bar{R}}_{0}$$

In S3 information retrieval, meanwhile, the data collection cost is the sum cost of local data collection and delivery of the aggregated data. Given *R̅_a_* as the average route length from each corresponding sensor node to the sink proxy, let *β* be the ratio of aggregated data size to the un-aggregated data size. Thus, the data collection cost in S3 information retrieval *E*_*C*3_ can be denoted by:

(7) $$E_{C3}=\alpha *N*E_{0}*{\bar{R}}_{a}+\beta *(\alpha *N)*R_{u}*E_{0}$$

Consequently, the ratio of data-collection cost of S3 to S2 is computed as:

(8) $$R_{C}=\frac{E_{C3}}{E_{C2}}=\frac{\alpha *N*E_{0}*{\bar{R}}_{a}+\beta *(\alpha *N)*R_{u}*E_{0}}{\alpha *N*E_{0}*{\bar{R}}_{0}}=\frac{{\bar{R}}_{a}+\beta *R_{u}}{{\bar{R}}_{0}}$$

In data collection operations, adopting an appropriate sink proxy consumes no larger energy than the conventional S2 approach. That is:

(9) $$R_{C}\leq 1$$

when *R_C_* ≤ 1 (*R*_0_ > *R̅_a_* + β*R_u_), there is energy saving in S3 data collection compared with S2 data collection.

This result indicates that the smaller *β* is, the smaller the relative cost of S3 becomes. The use of a sink proxy enables maximum aggregation of small sensor data and the smallest *β*, since all data are collected at the sink proxy and are aggregated before being transmitting to the sink.

*R̅*_0_, *R_u_* and *R̅_a_* can be calculated at the sink node based on the GPSR route discovery protocol that is described in [36], given the location of the sink proxy and resolved nodes corresponding to the query. *R̅*_0_ is a parameter that depends on the position of the corresponding sensor when using the shortest path routing. The values of *R_u_* and *R_u_* depend on the location of the sink proxy. As a result, the ratio of data-collection cost of S3 to S2 depends on the aggregation ratio and the position of sink proxy.

### 4.2. Impact on the Energy Bottleneck

With conventional S2 data collection, there is an energy bottleneck at the sink node. This occurs in multihop wireless sensor networks because most of the data are accumulated at the sink node, and nodes near the sink node are likely to consume more energy than nodes far removed from the sink node. The impact of the S3 model is that data are aggregated to the maximum degree before being delivered to the sink node and the sink proxy is selected from various sensor nodes, so that energy consumption is much balanced.

We analyze the impact of the S3 model on the energy bottleneck, with a specific focus on the energy reduction at the one-hop neighbour nodes of the sink node. Given a sensor network consisting of N nodes, and average energy consumption of a one-hop transmission of a packet assumed to be *E*_0_. Note *α* is the ratio of sensor nodes that will respond to a query message. Then, energy consumption *E*_*S*2_(1*hop*) at one-hop neighbour nodes of the sink in the conventional S2 model can be denoted as:

(10) $$E_{S2}\;(1\mathrm{hop})=\alpha *N*E_{0}$$

where *α* * *N* is the number of sensor nodes corresponding to a query. The energy consumption at the one hop neighbour nodes of sink in S3 model is denoted as:

(11) $$E_{S3}\;(1\mathrm{hop})=\alpha *N*E_{0}*\beta +\gamma *E_{0}$$

where *β* is the ratio of aggregated data size to un-aggregated data size, *γ* * *E*_0_ is the energy consumed at one-hop nodes for relaying a packet from a sensor to the sink proxy before collecting data at the sink. When the network is large, the number of one-hop nodes is small compared with the number of nodes in the network, the relay probability is very small, and *γ* * *E*_0_ can be ignored, and so:

(12) $$E_{S3}\;(1\mathrm{hop})\dot{=}\alpha *N*E_{0}*\beta$$

This means that energy consumption is *β* times the S2 model (*β* ≤ 1). The greater the aggregation capability of the network, the smaller the *β* is, leading to more energy savings. For example, when 10 packets each with 5 bytes of payload data are aggregated into one packet with a 10 × 5 payload, sharing one header with 28 bytes, *β* is (10 × 5 + 28)/(5 + 28) × 10 = 25%.

## 5. Simulation Evaluation and Numerical Results

### 5.1. Evaluation Metric, Objects, and Simulation Setup

We evaluate our proposed protocols using an NS-2 simulator, which is integrated with the IEEE 802.15.4 based MAC module [12,13]. In the simulation description, we call conventional on-demand approaches S2 (Sink-sensors), and our proposed approach S3 (sinl-sink_proxy-sensors).

The following protocols are evaluated in the simulation:

Flooding: A S2 approach with flooding based query dissemination and many-to-one data collection from reverse paths of the flooding [3].
LBM based information retrieval: A S2 approach with location-based multicasting for query dissemination and data collection from reverse paths of multicasting [20].
LDQD: Localised Data Query Distribution in S3 approach.
LSDC: Localised Sensing Data Collection in S3 approach.

LDQD and LSDC schemes are classified according to the position of the sink proxy. For instance, schemes that choose the centre node in the query area as a sink proxy are called LDQD centre and LSDC centre; schemes that use the nearest node in the query area as a sink proxy are called LDQD sink; schemes that use the random selected sink proxy in the query area are called LDQD limited random and LSDC limited random, schemes that use the network-wide random selected sink proxy are called LDQD network random and LSDC network random, *etc.*

The protocols are evaluated using the following metrics:

*Ratio of energy consumption*: The ratio of energy consumption for the proposed protocol S3 compared with conventional approaches S2, which includes flooding-based and LBM based protocols. It is defined as: $R_{\mathrm{ratio}}=\frac{E_{S3}}{E_{S2}}$; where *E*_*S*2_ is the energy consumption of conventional approaches of on-demand information retrieval (including Flooding and LBM); and *E*_*S*3_ is the energy consumption of proposed approaches of on-demand information retrieval.

*Energy consumption at each node*: The energy consumption at each sensor node during the simulation.

**Figure 5.**
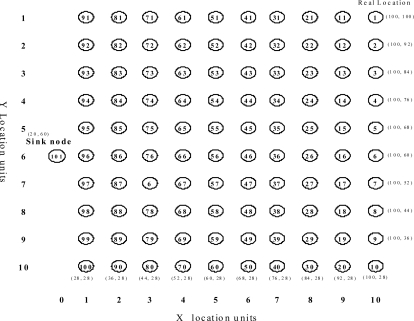
Simulation topology.

Unless otherwise stated, the simulation is set up as follows. There are 100 sensor nodes and 1 sink node in the network. The sensor nodes are distributed over a square of 100 m × 100 m, and the detailed topology of the sensor network is shown in Figure 5. The network topology is static and all nodes are connected to each other by either single-hop or multi-hop links. Each sensor node is equipped with an IEEE 802.15.4 radio module, and has a radio range of 15 m. The maximum number of sensing data that can be aggregated in a packet is set to 10.

### 5.2. Numerical Results

We first study the total energy consumption and data delivery ratio in sensor networks. To evaluate the information retrieval operation with various queries from users, in the simulation we adopt the variable of square areas in which users query sensors for data. The selection of the area being queried is defined by squares of different dimensions. The position of each square in relation to the corresponding nodes is randomly selected among the network. The total energy consumption in a round of data query and collection is the average of 10 cycles of data query and collection.

**Figure 6.**
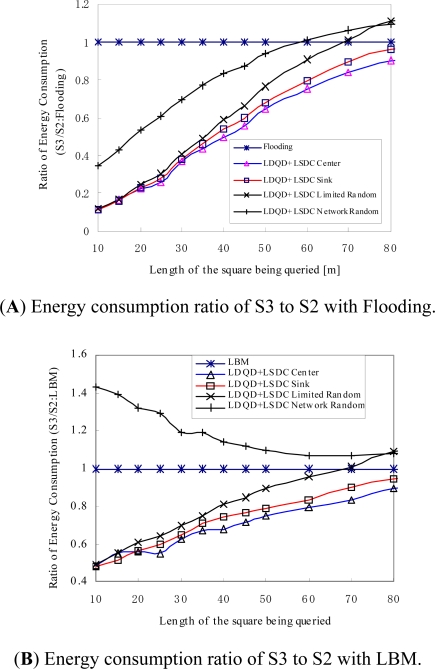
Ratio of energy consumption for approaches using S2 and S3 models.

Figures 6 (**A**) and (**B**) present the ratio of energy consumption in the proposed S3 based approaches to the conventional S2 based protocols, which include Flooding and LBM. Most of S3 based approaches, except for LDQD + LSDC Network Random approach, highly reduce the energy consumption compared with S2 approaches, especially when the length of the square being queried is small. In the S3 based approaches, the LDQD + LSCD Center approach that adopts the centre node of the query area as the sink proxy achieves optimised performance when the length of the square being queried is larger than 20, with a ratio of 25–90% energy consumption compared to the flooding-based protocol and 50–90% compared to the LBM-based protocol. The smaller the length of the square being queried, the larger the ratio of energy savings for the proposed approach. The exception is the LDQD + LSDC Network Random, which has greater energy consumption than LBM when the length of square is small because the network-wide random selection of a sink proxy cannot achieve a localised query and data collection for small query areas.

In addition to the evaluation of total energy consumption in the network, we studied the distribution of energy consumption at individual sensor nodes. To evaluate the impact of dynamic sink proxy rotation on the energy consumption among individual sensor nodes, we set up a simulation consisting of 40 identical queries with and each query was followed by a data collection operation. The length of query square is set to 40 m and the maximum number of sensing data that can be aggregated in a packet is set to 20.

**Figure 7.**
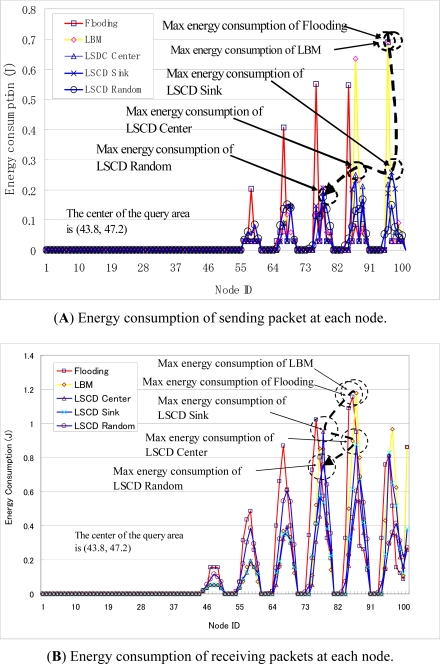
Energy consumption at each sensor node in data collection.

Figures 7 (**A**) and (**B**) shows an example of the distribution of energy consumption at each sensor node in the data collection operation. Flooding-query based data collection and LBM based data collections have high and non-uniform energy consumption with respect to both sending and receiving data. LSDC-center and LSCD-sink based data collections have much lower energy consumption at sensor nodes. LSCD-Random, in which the sink proxy is random selected in every query and rotates among sensor nodes, has the lowest value of the max energy consumption among sensor nodes. Hence, it has most uniform energy distribution among sensor nodes.

We also set up a simulation consisting of three query objects, which are selected in the network. Sensor nodes monitoring each object are located within a 20 m × 20 m square, in which effective sink proxy can be obtained. For each query object, 40 rounds of queries and data collections are performed. In the simulation S2 approach adopts LDQD + LSDC Limited Random with effective sink proxies.

**Figure 8.**
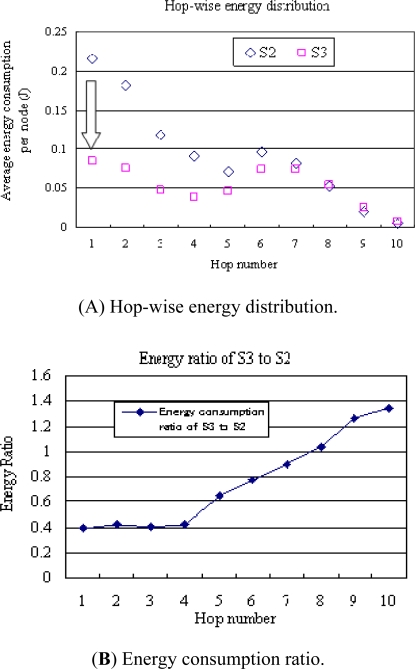
Energy consumption with regard to hop-distance.

Figure 8 (**A**) illustrates the energy consumption at nodes with respect to their hop-distances to the sink. In the conventional S2 model, nodes of one-hop neighbours of the sink have the largest energy consumption, far larger than that of nodes with a long distance to the sink. In the S3 model, the energy consumption was about 40% of that of the S2 model at nodes of one-hop neighbours of the sink, leading to significant alleviation of the energy bottleneck. Figure 8 (**B**) shows the energy consumption ratio of the S3 model to the S2 model. Energy consumption at nodes near the sink is significantly reduced using the S3 model. On the other hand, energy consumption at nodes located at a far distance is larger using the S3 model, leading to a more balanced energy distribution compared with the conventional S2 model. This is because the S3 model utilises dynamic sink proxies to balance energy consumption, and the sink node’s operations are distributed to sensor nodes.

**Figure 9.**
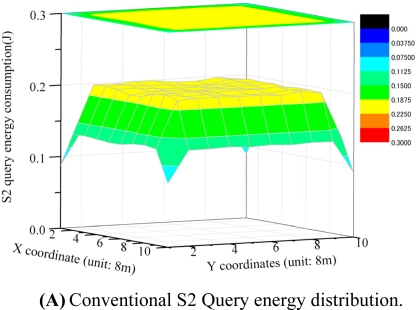

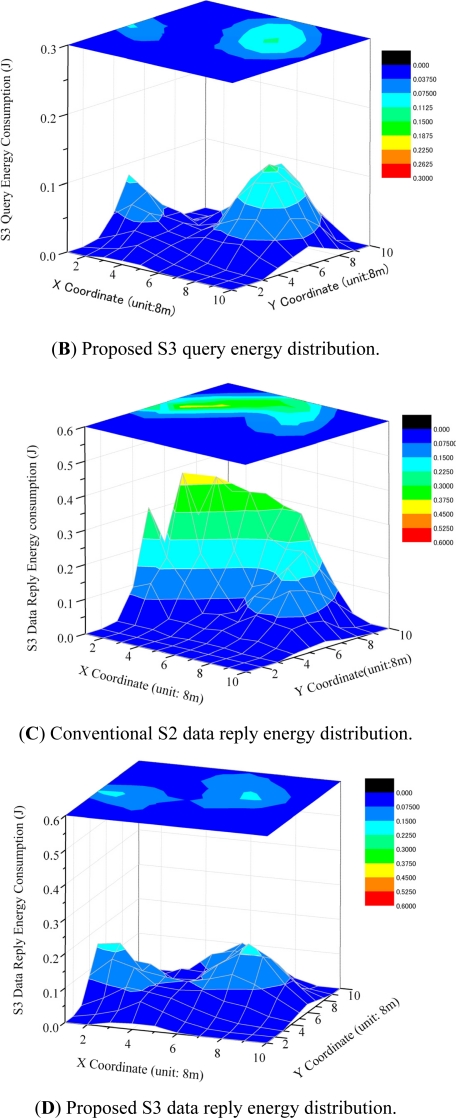
Energy consumption distribution among nodes.

Figures 9 (**A**) and (**B**) show the energy consumption of data queries at each sensor node. S2 queries have high energy consumption at most sensor nodes. The proposed S3 based query has much lower energy consumption for most sensor nodes. The largest energy consumption of the S3 query among sensor nodes is about 0.13 J, which is much smaller than that of S2 (0.2 J).

Figures 9 (**C**) and (**D**) show the distribution of energy consumption among sensor nodes in the data collection (reply) operation. S2-based data collection has high and non-uniform energy consumption at sensor nodes. Nodes near the sink node have the highest energy consumption: up to 0.42 J. In contrast, S3-based data collection has much lower energy consumption at sensor nodes. The highest energy consumption for S3 is 0.17 J, which is about 40% of that in S2-based data collection. Further, S3 data collection has more uniform energy distribution among sensor nodes.

## 6. Conclusions

This paper has proposed an on-demand localised information retrieval scheme for sensor networks with awareness of a user query’s content. In the proposed scheme, a query’s name is resolved into the IDs and locations of corresponding sensor nodes before being distributed to the network. According to the location of sensor nodes, query distribution and data collection are performed in a corresponding local area. The query message is efficiently unicasted to the sink proxy in a query area, and is then forwarded to a localised area of the network. Sensing data are collected at a sink proxy, at which data are aggregated and sent to the sink node. We provided an analytical model to describe the criteria of sink proxy selection and we analyzed the impact of the proposed approach on alleviating the energy bottleneck in data collection. The simulation results show that the proposed scheme significantly reduces the energy consumption of data query and data collection in the network. And the energy consumption is more uniformly distributed among sensor nodes than in conventional approaches of on-demand information retrieval. The proposed scheme achieves 60% of energy reduction at the neighbouring nodes of the sink in the simulation.

From the evaluation results, we know that the proposed scheme is promising for applications where sensing data are queried on-demand from users with diverse interests. As for future work, the in-network and high-level context abstracting of sensing data are considered. The maximised data aggregation in the proposed information retrieval model is expected to provide high effectiveness of context abstracting at sensor nodes.
